# Factors Associated with Multibacillary Leprosy in a Priority Region for Disease Control in Northeastern Brazil: A Retrospective Observational Study

**DOI:** 10.1155/2019/5738924

**Published:** 2019-02-18

**Authors:** Maria Aparecida Alves de Oliveira Serra, Crislane da Silva Santos, Pedro Martins Lima Neto, Karyne Gleyce Zemf Oliveira, Francisca Jacinta Feitoza de Oliveira, Ariadne Siqueira de Araujo Gordon, Daniella Pontes Matos, Raina Jansen Cutrim Propp Lima, Janaina Miranda Bezerra, Ismália Cassandra Costa Maia Dias, Floriacy Stabnow Santos, Ana Cristina Pereira de Jesus Costa, Marcelino Santos Neto, Antônio Rafael da Silva, Márcio Flávio Moura de Araújo

**Affiliations:** ^1^Universidade Federal do Maranhão, Imperatriz, Maranhão, Brazil; ^2^Programa de Pós-Graduação em Saúde e Tecnologia, Imperatriz, Maranhão, Brazil; ^3^Instituto Federal do Maranhão, Imperatriz, Maranhão, Brazil; ^4^Universidade da Integração Internacional da Lusofonia Afro-Brasileira, Redenção, Ceará, Brazil

## Abstract

**Background:**

Leprosy is an infectious disease that can lead to physical disabilities and stigmatization. It remains an important public health problem, especially in Brazil.

**Objective:**

To analyse sociodemographic and clinical factors associated with multibacillary leprosy in a hyperendemic region of the disease in northeastern Brazil.

**Method:**

This is a retrospective observational study with secondary data acquired from 2012 to 2015, from a group of leprosy cases reported in a reference outpatient clinic for the treatment and followup of leprosy in the city of Imperatriz, Maranhao, in northeastern Brazil.

**Results:**

From 905 new cases of leprosy studied, 656 (72.5%) were classified as multibacillary leprosy and 249 (27.5%) as paucibacillary leprosy. We observed that men were more likely to present 5 to 15 skin lesions (OR: 1.32; 95% CI: 1.18-1.49; p <0.0001) and >15 skin lesions (OR: 1.26; 95% CI: 1.09 -1.45; p = 0.005) and a lower chance of having <5 skin lesions (OR: 0.67; 95% CI: 0.59-0.76; p <0.0001). Women were more likely to have no affected nerves compared to men (OR: 1.46; 95% CI: 1.20-1.77; p <0.0001). The age range of 16 to 60 years showed a greater chance of having <5 skin lesions (OR: 1.01; 95% CI: 1.007-1.20; p = 0.03) and a lower chance of having 5 to 15 skin lesions (OR: 1.12, 95% CI: 1.03-1.23; p= 0.008) and a lower chance of being a grade I disability ( CI= 0.73-0.94; p=0.83) and II (OR: 0.82; 95% CI: 0.77-0.98; p=000.1).

**Conclusion:**

Cases of multibacillary leprosy were associated with male gender, low educational level, and clinical variables such as number of skin lesions and grade I or II disability.

## 1. Introduction

Leprosy is an infectious disease that can lead to the appearance of physical disabilities (dermatoneurological lesions) that result in stigmatization. It remains an important public health problem, especially in Brazil [1].

Despite the efforts of national and international health agencies to eliminate leprosy, this infectious disease is still an important public health problem that mainly affects populations living in unfavourable environments [2, 3].

Brazil ranks second worldwide in newly detected cases, preceded only by India [4]. Although some regions of the country have reduced or eliminated the disease, the North, Centre-West, and Northeast regions still present a high incidence and prevalence of the disease [5, 6].

The State of Maranhao, located in the Northeast region of Brazil, still exhibits a high endemicity pattern despite an incidence reduction in recent years. For example, Imperatriz city, the second largest socioeconomic and state political centre, ranks 13^th^ among Brazilian cities in the number of new cases of leprosy, with an incidence rate of 73.87 per 100,000 inhabitants. This number classifies the city as a hyperendemic region [7, 8].

The detection of leprosy cases is based on a range of clinical manifestations, and cases are classified for therapeutic purposes as paucibacillary (PB) and multibacillary (MB). The latter classification is related to a cellular immune response that is not effective in the control of* M. leprae*, leading to the development of aggressive forms of the disease, characterized by high transmissibility and high number of functional disabilities [9, 10].

A high incidence of MB leprosy and disabilities related to this disease may reflect deficits in a country's health system with respect to epidemiological surveillance and health education, resulting in late diagnosis [4, 11, 12].

We believe that information about clinical and sociodemographic variables of populations affected by leprosy add to the epidemiological surveillance of this disease. In hyperendemic regions, this information is even more crucial to clarify which groups and territories are more vulnerable and which interventions should be prioritized. This knowledge is useful for the creation of truly population-centered guidelines and for active case-finding.

Therefore, the objective of this study was to analyze the sociodemographic and clinical factors associated with MB leprosy in a hyperendemic region of the disease in northeastern Brazil.

## 2. Methods

### 2.1. Design and Location of the Study

The research was approved by the Committee of Ethics in Research with Human Beings of the Federal University of Maranhao according to opinion number 870.507. This is an observational, retrospective study using secondary data acquired from 2012 to 2015.

The research was conducted from April to June 2016 in a reference outpatient clinic for the treatment and followup of leprosy cases in the city of Imperatriz, Maranhao, northeastern Brazil.

This city has the second largest population and is a socioeconomic, political, and cultural centre of the State of Maranhao (ranked third in number of leprosy cases in Brazil) with a high human development index (0.731). Nevertheless, the region still faces problems regarding illiteracy rates (9.7%), basic sanitation (23%), and domiciliary drinking water (86%). The city is located 626 km from Sao Luis (with the largest number of new leprosy cases in the country), which is the capital of the state, and has a territory of 1,368.98 km and a population of approximately 252,320 inhabitants [8, 13].

### 2.2. Data Collection

In the Brazilian primary and secondary health care services, a specific notification form is required for each new case of leprosy. These data are then recorded in the National System of Notifiable Diseases (Sistema Nacional de Agravos Notificaveis-SINAN).

Data collection in this study was conducted from these records of notification of diagnosed cases of leprosy from 2012 to 2015 at the aforementioned location. The variables investigated included sex, age, education, occupation, skin colour, number of contacts, number of skin lesions, clinical form, number of nerves affected, smear microscopy, and degree of disability at the beginning of treatment. Incomplete data sheets (n = 7) were excluded from the sample.

We have chosen the multibacillary leprosy operational classification and the sociodemographic and clinical factors mentioned previously as the outcome variable and predictors, respectively. The classification of multibacillary leprosy followed the criteria adopted by the Brazilian Ministry of Health in the analysed period (it includes a dimorphic or lepromatous clinical form and/or positive bacilloscopy) [14].

These criteria have been conducted by medical and epidemiological records and dermatological and neurological exam. Detection of acid-fast bacilli in a skin smear in a patient categorizes that a person has the MB form of the disease. A negative result, however, does not exclude the disease (leprosy) as well as does not indicate PB case. It is important to understand that when more than one nerve is affected (at dermatological and neurological exams) the individual is characterized as having the MB form regardless of the number of skin lesions [14].

### 2.3. Data Analysis

Data processing and statistical analysis were performed using the* Statistical Package for Social Science ®*, version 22.0 (SPSS 22.0, IBM, Armonk, NY, USA). Quantitative variables are presented as descriptive statistics (mean and standard deviation), and qualitative variables are presented as proportions and 95% confidence intervals.

We have organized the analysis variables into categories for statistical purposes. We performed the* Kolmogorov-Smirnov *and* Levene* tests to assess normality and homoscedasticity of the quantitative variables, respectively. The* Pearson's chi-square test* was used to analyse the effects of factors and the results are expressed as odds ratios (sociodemographic versus clinical factors). The significance level was p < 0.05. Logistic regression analysis was used to adjust for confounding variables to determine the independent risk factors for the MB form of leprosy.

To determine which independent risks are related to the operational classification of leprosy, we have used a forwards stepwise logistic regression method. This approach fits a model designed for a dichotomous dependent qualitative variable (the operational classification of leprosy) to analyse the significance of each of the independent variables. The resulting logistic regression model was used to classify subjects as PB or MB cases. The percentage of correct identifications was 72.6%. This number was higher than the proportional percentage of correct classifications by chance (50%) demonstrating the utility of the model to classify new observations.

## 3. Results

A total of 912 records of new cases of leprosy were considered, of which 905 had complete data and were included in this study. Of these cases, 59.9% were males, aging from 4 to 98 years (mean age: 43.1, SD ± 19.1), 10.4% were younger than 15 years of age, 19.7% were 60 or older, 68% had brown skin, 78.1% had <10 years of schooling, 58.9% were employed, and 84.5% had <5 close contacts. Most patients (72.5%) were classified as multibacillary.

We observed that men were more likely to present 5 to 15 skin lesions (p <0.0001; OR: 1.32; 95% CI: 1.18-1.49) and >15 skin lesions (p = 0.005; OR: 1.26; 95% CI: 1.09 -1.45) and a lower chance of having <5 skin lesions (p <0.0001; OR: 0.67; 95% CI: 0.59-0.76). Women were more likely to have no affected nerves than men (p <0.0001; OR: 1.46; 95% CI: 1.20-1.77).

The age range of 16 to 60 years showed a greater chance of having <5 skin lesions (OR: 1.01; 95% CI: 1.007-1.20; p = 0.03) and a lower chance of having 5 to 15 skin lesions (OR: 1.12, 95% CI: 1.03-1.23; p = 0.008) and a lower chance of being a grade I disability (95% CI 0.73-0.94; p = 0.830).

### 3.1. Multibacillary Leprosy and Sociodemographic Factors

The following groups were found to have a greater likelihood of being classified as multibacillary: males, aged ≥ 60 years of age, with <10 years of education, employed individuals, and retirees (Table 1).

After adjusted logistic regression analysis, male gender (OR = 2.95, 95% CI = 2.12–4.11; p < 0.0001) and low education level (OR = 1.71, 95% CI = 1.18–2.46; p = 0.004) remained significantly associated with multibacillary leprosy.

A lower likelihood of multibacillary leprosy was found in unemployed individuals and children, Table 1. After adjusted logistic regression analysis, these associations were not statistically significant.

### 3.2. Multibacillary Leprosy and Clinical Factors

Of the 72.5% cases of multibacillary leprosy, 40.8% had less than five skin lesions, 51.8% had no peripheral level nerves, and 38.9% had zero disability (Table 2).

We observed that patients with less than five skin lesions were less likely to be classified as multibacillary compared to those with 5-15 and > 16 skin lesions, Table 2.

Among the patients with affected peripheral nerves, those with affected nerves, positive skin smear microscopy, or a neurological disability degree of I or II were more likely to be classified as multibacillary (Table 2).

The reported patients who had zero disability were less likely to have multibacillary leprosy (Table 2).

After adjusted logistic regression analysis, the clinical variables that remained associated with multibacillary leprosy included having 5-15 skin lesions (OR = 7.18, 95% CI = 1.04–49.1; p = 0.04) and grade I disability (OR = 7.96, 95% CI = 1.21–52.2; p = 0.03) or II (OR = 16.4, 95% CI = 1.85–146.8; p = 0.01).

## 4. Discussion

Findings from this study reflect the hyperendemicity of the studied region, where late diagnosis of leprosy still occurs, leading to the development of severe forms of the disease and disabilities [15, 16].

The men have presented worse clinical prognosis. In fact, this research reveals that males are more affected by cases of MB form of leprosy compared to the PB form, even after model adjustment. This result is similar to what was found in other regions of Brazil [17–19] and in countries such as Ethiopia [20], Mexico [21], and the United States [22]. These data suggest that males could be more exposed to the bacillus, which is reflected by behavioural and cultural factors [19, 23].

It is important to make some considerations since we have conducted this research in Brazil. In this country, leprosy primary health care services do not work overnight; this fact compromises adherence of the masculine population (in job). Besides that, cases were recorded in environments with large male agglomerations such as prisons or an army. These points could partially explain about a predominance of cases among men.

There is also a possible relation between male hormones and the occurrence of MB form of the disease, since male hormones stimulate immune responses that are less effective for the disease control, especially during puberty [24, 25].

Our findings could also be explained by the fact that intensive active search of leprosy cases are usually held at places that are mainly visited by men, such as military quarters and prisons. In addition, the findings could be a consequence of active home-search strategies that are common in Brazil. Women usually visit primary care services more frequently than men, and they may request a home visit from a health worker if they suspect that their husbands have leprosy.

The greater vulnerability of the elderly to multibacillary leprosy is likely related to the prolonged incubation period of the Hansen bacilli and, consequently, late manifestation [19, 26]. In addition, the immunological senescence of the elderly is an aggravating factor for infection control [27, 28]. Consequently, the association between multibacillary leprosy and retirees (widely represented by the elderly) confirmed in this study is understandable. In Brazil, this is worrying because in two decades the country will have the largest population of elderly people in the world in absolute numbers.

A previous study showed that there is a shift in the incidence of leprosy, from the PB to MB form, from young to older adults (especially those aged from 60 to 69 years), and from females to males (almost twice more susceptible to leprosy) [19].

Even after model adjustments, a low educational level was associated with the multibacillary form of leprosy. Authors agree that increased education contributes to adequate knowledge of the signs and symptoms of diseases, leading to a better understanding of individual health and daily life. Thus, highly educated individuals tend to avoid delays in seeking health services [16, 29, 30].

A predominance of the multibacillary form was observed among employed individuals, which probably suggests that working conditions favour exposure to the bacillus, but we cannot rule out the importance of social contacts in the transmissibility of the disease [31, 32]. A previous Brazilian study concluded that the transmission of leprosy is expanding and is not restricted to the home environment. Thus, urban spaces are also important for the spread of the disease [33].

Cases with 5-15 skin lesions remained associated with multibacillary leprosy after model adjustments, demonstrating that high bacillary loads contribute to greater tissue destruction and, consequently, deforming skin lesions (responsible for stigmatization). A study that analysed cutaneous lesions in patients with leprosy showed that innate immunity factors, such as activation of complement proteins, were significantly greater in multibacillary patients than in those with paucibacillary skin lesions, which can potentiate the inflammatory process and contribute to peripheral nerve injury in multibacillary cases [34].

The degree of disability has been used as an indicator of the capacity of the health services to diagnose and monitor leprosy cases. Patients classified with a disability grade I or II are associated with a late diagnosis or failure to monitor cases [4, 11, 12].

The multibacillary cases in this study had more chances of having grade I or grade II disabilities, even after model adjustment. By adding disability grade I (n = 193, 21.3% of the total) to disability grade II (n = 96, 10.6%) the number of patients with disabilities increases to 289 (32%), which represents a high percentage of overall disability. In addition, the incidence of grade II disability in our sample is much higher than the 6% worldwide average reported by the WHO, in 2016, which suggests delayed diagnosis or misdiagnosis in this sample of patients.

This study has limitations typical of studies based on secondary data such as lack of uniformity in information collection, prevalence bias, and cross-sectional design. Thus, future research with a longitudinal or geographical distribution design is needed to elucidate the factors associated with the infection. The data obtained in these studies may contribute to the effective prevention and control of leprosy, based on the epidemiologic and territorial characteristics.

## 5. Conclusion

In the sample studied, cases of multibacillary leprosy were associated with male gender, low educational level, and clinical variables such as number of skin lesions and grade I or II disability.

## Figures and Tables

**Table 1:** Association between sociodemographic variables and forms of leprosy.

Operational Classification					
Variables	Paucibacillary N = 249	Multibacillary N = 656	P-value	OR	95% CI
	*N (*%)	*N (*%)			
*Sex*					
Male	97 (38.9)	445 (67.8)	<0.0001	1.41	(1.28-1.55)
Female	152 (61.1)	211 (32.2)			

*Age*					
< 15 years	36 (14.4)	58 (8.9)	0.01	0.83	(0.71-0.98)
16-60 years	176 (70.6)	457 (69.6)	0.76	0.98	(0.90-1.07)
> 60 years	37 (15)	141 (21.5)	0.02	1.11	(1.02-1.22)

*Education*					
≤ 10 years of education	172 (69.1)	541 (82.5)	<0.0001	1.26	(1.12-1.43)
> 10 years of education	77 (30.9)	115 (17.5)			

*Occupation*					
Employed	132 (53)	401 (61.1)	0.02	1.09	(1.009-1.19)
Unemployed	86 (34.5)	141 (21.5)	<0.0001	0.81	(0.73-0.91)
Retired	28 (12.5)	117 (17.4)	0.01	1.13	(1.03-1.24)

*Skin Colour*					
White	57 (22.9)	121 (18.4)	0.13	0.92	(0.82-1.03)
Black	24 (9.6)	88 (13.5)	0.12	1.09	(0.98-1.22)
Brown	168 (67.5)	447 (68.1)	0.84	1.009	(0.92-1.10)

*No. of contacts*					
≤ 5	209 (83.9)	556 (84.7)	0.76	1.01	(0.98-1.14)
> 5	40 (16.1)	100 (15.3)			

**Table 2:** Association between clinical variables and forms of leprosy.

Operational Classification					
Variables	Paucibacillary N = 249	Multibacillary N = 656	p-value	Or	95% CI
	*N (*%)	*N (*%)			
*Skin Lesions*					
No lesions	26 (10.4)	55 (6.7)	0.33	0.93	(0.79-1.08)
< 5 lesions	200 (80.3)	268 (40.8)	<0.0001	0.64	(0.59-0.70)
5 to 15 lesions	11 (9.3)	203 (30.9)	<0.0001	1.44	(1.35-1.54)
> 16 lesions	0 (0.00)	142 (21.6)	<0.0001	1.48	(1.41-1.56)

*Affected Nerves*					
No nerves	194 (77.9)	340 (51.8)	<0.0001	3.27	(2.34 - 4.58)
Affected nerves	55 (22.1)	316 (48.2)			

*Degree of Disability*					
Degree 0	142 (57)	294 (44.8)	0.001	0.87	(0.80- 0.94)
Degree I	28 (11.2)	165 (25.1)	<0.0001	1.24	(1.14- 1.33)
Degree II	14 (5.3)	82 (12.7)	0.003	1.20	(1.09-1.32)
Not evaluated	66 (26.5)	114 (17.4)	0.002	0.84	(0.75-0.95)

## Data Availability

The data used to support the findings of this study are available from the corresponding author upon request.
